# Manipulation of tissue factor-mediated basal PAR-2 signalling on macrophages determines sensitivity for IFNγ responsiveness and significantly modifies the phenotype of murine DTH

**DOI:** 10.3389/fimmu.2022.999871

**Published:** 2022-09-12

**Authors:** Hannah Wilkinson, Hugh Leonard, Michael G. Robson, Richard Smith, ElLi Tam, John H. McVey, Daniel Kirckhofer, Daxin Chen, Anthony Dorling

**Affiliations:** ^1^ Department of Inflammation Biology, School of Immunology & Microbial Sciences, King’s College London, Guy’s Hospital, London, United Kingdom; ^2^ School of Bioscience & Medicine, Faculty of Health and Medical Sciences, University of Surrey, Guildford, United Kingdom; ^3^ Department of Early Discovery Biochemistry, Genentech Inc., South San Francisco, CA, United States

**Keywords:** innate immunity, protease (proteinase)-activated receptor, type IV hypersensitivity, macrophage, thrombin

## Abstract

**Background:**

Tissue factor (TF) generates proteases that can signal through PAR-1 and PAR-2. We have previously demonstrated PAR-1 signalling primes innate myeloid cells to be exquisitely sensitive to interferon-gamma (IFNγ). In this work we explored how TF mediated PAR-2 signalling modulated responsiveness to IFNγ and investigated the interplay between PAR-1/-2 signalling on macrophages.

**Methodology:**

We characterised how TF through PAR-2 influenced IFNγ sensitivity *in vitro* using PCR and flow cytometry. and how it influenced oxazolone-induced delayed type hypersensitivity (DTH) responses *in vivo*. We investigated how basal signalling through PAR-2 influenced PAR-1 signalling using a combination of TF-inhibitors and PAR-1 &-2 agonists and antagonists. Finally, we investigated whether this system could be targeted therapeutically using 3-mercaptopropionyl-F-Cha-Cha-RKPNDK (3-MP), which has actions on both PAR-1 and -2.

**Results:**

TF delivered a basal signal through PAR-2 that upregulated SOCS3 expression and blunted M1 polarisation after IFNγ stimulation, opposing the priming achieved by signalling through PAR-1. PAR-1 and -2 agonists or antagonists could be used in combination to modify this basal signal *in vitro* and *in vivo*. 3-MP, by virtue of its PAR-2 agonist properties was superior to agents with only PAR-1 antagonist properties at reducing M1 polarisation induced by IFNγ and suppressing DTH. Tethering a myristoyl electrostatic switch almost completely abolished the DTH response.

**Conclusions:**

TF-mediated signalling through PARs-1 and -2 act in a homeostatic way to determine how myeloid cells respond to IFNγ. 3-MP, an agent that simultaneously inhibits PAR-1 whilst delivering a PAR-2 signal, can almost completely abolish immune responses dependent on M1 polarisation, particularly if potency is enhanced by targeting to cell membranes; this has potential therapeutic potential in multiple diseases.

## Introduction

Coagulation proteases, which mediate cellular responses by signaling through protease activated receptors (PARs), play a major role in the pathophysiology of, and is the leading cause of death in severe sepsis (1). This has most recently highlighted on a global scale by the COVID-19 pandemic, in which a prothrombotic state in response to the Sars-COV-2 virus has been reported to lead to widespread, sometimes catastrophic, intravascular coagulation (2). This phenomenon can be seen in all severe bacterial and parasitic infections (3).

Central to the generation of coagulation proteases in these settings is tissue factor (TF) expressed by bone marrow derived leukocytes (as well as platelets) (4). TF is a transmembrane receptor which exists in two forms on the surface of myeloid cells – described as ‘decrypted’ or ‘encrypted’ TF. ‘Decrypted’, or pro-coagulant TF acts an enzymatic cofactor by stabilizing the active conformation of the FVIIa protease domain and by contributing to an extended recognition interface for FX (5). The resulting activation of FX leads ultimately to the generation of thrombin which is then able to signal through PAR-1 (6). In the resting state TF exists in an ‘encrypted’ configuration, here TF is unable to mediate the generation of FXa. This encrypted TF is, however, able to signal through PAR-2, although this pathway is less well defined (7). What precisely controls this TF encryption/decryption “switch” remains to be determined (8).

We have previously demonstrated that thrombin, signalling through PAR-1, primes macrophages to be exquisitely sensitive to low concentrations of IFNγ (and TLR-4 stimuli) *via* reduced expression of the reverse cholesterol transporter ABCA1 and consequent increase in the number of membrane cholesterol-rich micro-domains, into which enhanced numbers of IFNγR (and TLR4) are recruited (9). We showed this mechanism was highly relevant in two *in vivo* murine models not usually associated with thrombosis: established atherosclerosis in ApoE-/- mice fed a high fat diet (10) and cutaneous delayed type hypersensitivity (DTH) (9). In both models, inhibiting thrombin-mediated signalling through PAR-1 on myeloid cells by transgenic expression of a tethered thrombin inhibitor led to profound phenotypic changes, including clearance of established atherosclerotic plaques in ApoE-/- mice and near complete inhibition of swelling and granuloma formation in DTH responses, through with preservation of T cell sensitisation. Both pieces of work clearly implicated thrombin mediated signalling on monocytes/macrophages as an essential determinant of the phenotype of the inflammatory response in these models.

In both models, we also reported that IV administration of a novel thrombin inhibitor called PTL060 (Thrombalexin) (11–13), could induce the same phenotype in ApoE-/- and WT mice respectively as that seen in transgenic mice (9, 10). Importantly, PTL060 binds rapidly to circulating cells after IV administration, including monocytes, on which we showed it exerts an extended pharmacological effect. Therefore, reversal of atherosclerosis was achieved using a dosing regimen in which animals were systemically anticoagulated for only 1/7^th^ of the time that PTL060 exerted its anti-inflammatory effect, effectively uncoupling, pharmacodynamically, the anti-inflammatory effects of inhibiting PAR-1 signalling on from the anticoagulant effects of systemic thrombin inhibition.

In contrast to the well-defined pro-inflammatory PAR-1 signalling outcome (14) the consequences of PAR-2 signalling are less well understood, in part because of conflicting reports in the literature. For instance, in models of DTH, both pro- (15) and anti- (16) inflammatory outcomes after PAR-2 signalling have been described. In the work presented here we investigate the role of PAR-2 signalling on macrophages *in vitro* and in the same murine DTH model described above. We also describe a novel therapeutic generated to exploit the highly novel findings we report; this has significant translational potential for clinically safe manipulation of these pathways to treat inflammatory diseases mediated by IFNγ-driven type IV hypersensitivity responses.

## Methods

### Animals

6–12-week-old male C57BL/6 mice were purchased from Envigo and housed in specific pathogen free environment. CD45.1 and CD31-Hir-Tg or CD31-TFPI-Tg (17) mice were bred in house on a C57BL/6 background. All procedures were performed in accordance with the Home Office Animals (Scientific Procedures) Act of 1986.

### Delayed type hypersensitivity experiments

On Day 0 a 50µl preparation of 5% oxazolone (Sigma, Dorset, UK) in ethanol and acetone (4:1) was applied to the shaved abdomen. Mice were re-challenged on day 5 by applying 1% oxazolone in olive oil and acetone (4:1, 10 µl) to the right ear and vehicle alone to the left ear. Ear thickness was measured using a digital micrometre using at least 5 measurements and this was subtracted from the mean ear thickness of the vehicle treated ear. After 48 hours the mice were anesthetised and sacrificed by cervical dislocation and the ears removed and added to a cryomold and covered in OCT. Samples were stored at -80°C prior to analysis. *In vivo* mice were treated with IP PAR agonists/antagonists at the molarity described in the experiments prior to rechallenge on day 5. For the PTL060 experiments mice received 10µg/g IV PTL060 on day 3 and day 5 (3 hours before re-challenge). Immediately after last IP injection, the mice were re-challenged with 1% oxazolone in olive oil and acetone (4:1, 10 µl) to the right ear and vehicle alone to the left ear. Anti-TF experiments: on day 4 after sensitisation with oxazolone mice received 10µg/g IP rat anti-mouse TF Ab 1H1 (18).

### Bone marrow transplant

Recipient mice were irradiated with 9 Gy and then reconstituted with 5x10^6^ bone marrow cells (see below for isolation protocol) intravenously *via* tail vein within 24 hours of irradiation. Mice were weighed daily and monitored for signs of distress. Engraftment was assessed by surface CD45.1 and CD45.2 expression on peripheral blood cells acquired through tail vein venepuncture by flow cytometry after day 30.

### Immunofluorescence analysis

Tissue sections were cut (5μm) using a cryostat (Bright Instrument Ltd, Huntington, UK) and transferred onto multispot glass slides (Hendley-Essex, Loughton, UK). Sections were fixed in methanol for 1 hour at -20°C and then left to air dry. Sections were then blocked with 10% foetal calf serum (FCS) in PBS for 1 hour after which they were washed 3 times for 5 minutes in PBS, 0.5% Triton X-100. Primary antibodies used in this study were rat anti mouse CD68 (FA-11 Thermofisher Scientific, UK) and CD3 (ab5690 Abcam, Cambridge, UK) and rabbit anti mouse CD206 (ab64693 Abcam), iNOS (ab15323 Abcam), ABCA1 (ab7360 Abcam), IL-10 (ab34843 Abcam) and IFNγ (ab9657 Abcam). Primary antibodies were incubated overnight in a humidified chamber, before washing and application of the secondary antibody (goat anti-rat AF594 or goat anti-rabbit AF488 (Abcam)) for 2 hours at RT. Slides were mounted with Vectashield Antifade Mounting Media with DAPI (2BScientific, Oxford, UK) and covered with glass cover slips. All sections were stored in the dark at 4 °C before analysis using a fluorescence microscope. For quantification, images were assessed at 100X magnification, background signal was assessed with isotype and no primary antibody controls. Using ImageJ software, the area of the lesion was drawn around and percentage expression assessed using threshold measurements to remove background signal. At least 3 sections were taken per mouse and 5 images were taken per section. Co-localisation analysis was performed using Pearson correlation analysis on ICY software.

### Peritoneal macrophage isolation

WT mice received 1 ml of 4% thioglycollate broth (Sigma-Aldrich) intraperitoneally. 72 hours later cells were harvested by peritoneal lavage with 3 ml of ice-cold HBSS and washed and resuspended in 1.2 ml PBS for flow cytometric analysis.

### ELISA

Anticoagulated whole mouse blood (EDTA 30 mmol/L pH8) was separated into plasma and cells by centrifugation (14 000g for 10 minutes). Plasma TNF-α, IFNγ, MIF, CCL2, CCL5 and CX3CL1 were detected using separate specific ELISA kits (Abcam, Cambridge, UK) according to the manufacturers’ instructions. Total cholesterol (TC), high-density lipoprotein (HDL) and low-density lipoprotein (LDL) and Triacylglycerol (TG) in plasma were determined with commercial kits (TC, HDL and LDL Kits, Cell Biolabs, Cambridge UK; and TG kit, Abcam) according to the manufacturer’s protocol.

### Bone marrow isolation protocol

Mice were euthanized by cervical dislocation. Bone marrow cell suspensions were isolated by flushing femurs and tibias of 8–12-week-old donor mice with Dulbecco’s Modified Eagle Medium (DMEM). Aggregates were dislodged by gentle pipetting, and debris was removed by passing the suspension through a 70-µm cell strainer. Isolated cells were counted and plated on a Nunc™ Non-Treated 6 well plate (Thermofisher Scientific) at 1x10^6 cells/ml in DMEM glutamax, high glucose, high pyruvate (Thermofisher Scientific) supplemented with 10% FCS, 1% non-essential amino acids, 1% penicillin/streptomycin and 2μM Mercaptoethanol (Thermofisher Scientific). To induce macrophage formation 25ng/ml macrophage colony-stimulating factor (MCSF) (Biolegend, London, UK) was added to the culture medium. Cells where then placed in a humidified incubator at 37°C at 5% CO_2._ Media was changed for fresh media every 48 hours and grown for 5 - 7 days.

### *In vitro* macrophage stimulation

Cells were used at 1x10^6 cells per ml in DMEM glutamax, high glucose, high pyruvate (Thermofisher Scientific) supplemented with 10% FCS, 1% non-essential amino acids, 1% penicillin/streptomycin and 2μM Mercaptoethanol, plated in 12 or 24 well plates. For titration experiments cells were exposed to increasing concentrations of lipopolysaccharide (LPS) or IFNγ. For full M1 polarisation cells were stimulated with 100ng/ml LPS (from Escherichia coli O55:B5-Sigma) and 50ng/ml IFNγ (Thermofisher Scientific), whereas canonical M2 polarisation was achieved by 25ng/ml IL-4 (BD Biosciences, Berkshire, UK). In some experiments, cells were primed with various concentrations of thrombin (Enzyme Research Lab, Swansea UK) for 24 hours prior to exposure to IFNγ, LPS or IL-4 (where thrombin remained in culture). All experiments occurred in 10% FCS containing media. Selected PAR agonists or antagonists, as described below, were used in some experiments.

### *In vitro* chemokine assay

Primary mouse smooth muscle cells (MSMCs) were purified as previously described (19) and seeded at a density of 1×10^6^ cells/well of a 24-well plate. They were serum-starved for 24 hours before addition of reagents. PAR-1 agonists (H-Ser-Phe-Leu-Leu-Arg-Asn-NH2 (SFLLRN-Amide) at 10 µM) or (H-Thr-Phe-Leu-Leu-Arg-NH2 (TFLLR-amide) at 10 µM), PAR-1 antagonist (H-Phe-Leu-Leu-Arg-Asn-OH (FLLRN) at 10 µM), PAR-2 antagonist(H-Phe-Ser-Leu-leu-Arg-Tyr-NH2 (FSLLRY-Amide) at 10 µM), PAR-2 agonist (2-Furoyl-Leu-lle-Gly-Arg-Leu-Orn-NH2 (2-Furoyl-LIGRLO-Amide) at 10 µM) or combined PAR-1 antagonist/PAR-2 agonist (3-mercaptopropionyl-F-Cha-Cha-RKPNDK (3-MP) at 10 µM) were all from Peptides International Inc. (Louisville, KY40269-0703, USA) and were incubated for 12 hours with the starved MSMCs. In some assays, thrombin (10 nM) (Enzyme Research Laboratories. Swansea, UK) was then added for 1 hour with 2% FCS DMEM. The medium was then changed, and cells incubated for a further 48 hours before the supernatants were collected to measure MIF, CCL2, CCL5 and CX3CL1 by ELISA using kits from Abcam.

### TF activity assay

MCSF-derived BMM were lysed in 15mM Octyl β-D-glucopyranoside for 15 minutes at 37°C then TF activity was measured by production of FXa using a specific TF activity assay (ab108906 ABCAM).

### ‘Cytotopic’ peptides

PTL060 is a direct thrombin inhibitor, comprising hirulog covalently linked to a synthetic myristoyl electrostatic switch to tether to cell membranes; it has been described elsewhere (10, 12). PTL0GC-1 comprises a PAR-1 antagonist -3-MP (20) tethered to the same synthetic myristoyl electrostatic switch for tethering to cell membranes. Briefly, it was formed by conjugating 3-MP with the cytotopic tail APT3098 [(N-a,N-d,bis-myristoyl Lys-Ser-Ser-Lys-Ser-Pro-Ser-Lys-Lys-Asp-Asp-Lys-Lys-Pro-Gly-Asp-LysBrAc]. The conjugate has a mass of 3617 Da by time-of-flight mass spectrometry. The component peptides and conjugates were prepared under contract using solid-phase synthesis by Almac Sciences, Craigavon, UK.

### Flow cytometry

All flow cytometry was performed on a Fortessa LSR II flow cytometer (Becton Dickinson) using DIVA software (Becton Dickinson) and analysed using Flow-jo (Treestar, Ashland, OR) software. Prior to surface staining cells were incubated with mouse Fc Block (Biolegend) for 5 minutes in the dark at 4°C, after which 50μL of the relevant antibody cocktail was added, and the cells were left to incubate in the dark at 4°C for 30 minutes. Surface antibodies were FITC – F4/80, APC- CD11b, PE-ABCA1 (Santa Cruz Biotechnology, Heidelberg, Germany) FITC-TF (Biorbyt, Cambridge, United Kingdom) or FITC-TF (American Diagnostica). After surface staining cells were resuspended in 200μl pre-diluted Near IR live/dead stain (Life Technologies) and left to incubate in the dark at 4°C for 15 minutes. For intracellular staining, cells were permeabilised with Foxp3 intracellular staining permeabilisation solution for 30 minutes (e-Bioscience). Intracellular staining was performed using directly conjugated antibodies (BV605-CD206 (Biolegend) and PE-Cy7-iNOS (e-Bioscience)) made up into a staining cocktail using permeabilisation buffer (e-Bioscience), 50μL of staining cocktail was added per well and staining took place at 21 °C in the dark.

### SiRNA

MCSF-derived bone marrow macrophages (BMM) were plated at 2x10^5 cells/ml in DMEM 10% FCS. 500 µL cell suspension was added to a 24 well plate. SiRNA was prepared using Lipofectamine™ RNAiMAX Transfection Reagent with Opti-MEM™ Reduced Serum Medium and Silencer™ pre-Designed siRNA (ThermoFisher Scientific). Cells were transfected in complete medium for 24 hours as per manufacturers instruction at 37°C, 5% CO_2_. FITC conjugated positive control SIRNA and negative control SIRNA was also used (sc-36869 and sc-37007 respectively, Santa Cruz). After 24 hours 50U/ml thrombin was added, and cells were further incubated for 24 hours at 37°C at 5% CO_2._ After this 24-hour incubation the cells were then washed and new media with LPS/IFNγ with or without thrombin was added for a further 24 hours. Cells were then analysed for ABCA1 and iNOS expression using flow cytometry as described above.

### RT PCR

Total RNA was extracted using RNeasy mini-Kit (Qiagen). RNA quantity was analysed by Nano drop system. Reverse transcription was carried out using the QuantiTect Reverse transcription Kit (Qiagen). Genomic DNA was eliminated using the provided gDNA wipe-out buffer. The PCR step was performed using TaqMan fast advanced master mix with TaqMan gene expression assays (ThermoFisher scientific). PCR assays used were: TBP (Mm01277042_m1), iNOS (Mm00440502_m1), TNFα (Mm00443258_m1), IL-1β (Mm00434228_m1), IL-6 (Mm00446190_m1) and RANTES (Mm01302427_m1). The plate was then set up on the BioRad CFX96 Real Time PCR detection system. The results were then normalised to the housekeeping gene TATA box binding protein (TBP) using the delta Ct method

Statistical analysis: All statistical analyses were performed using GraphPad Prism^®^ software version 7. Unpaired samples were compared using a Mann Whitney U test with two tailed p-values. One and two way ANOVA for multiple comparisons. P-values are shown as * P <0.05 ** P < 0.01 *** P < 0.001 **** P < 0.0001.

## Results

### PAR-2 signaling reduces macrophage sensitivity to IFNγ *via* increased expression of SOCS3 and inhibits acute DTH responses

**Figure 1:**
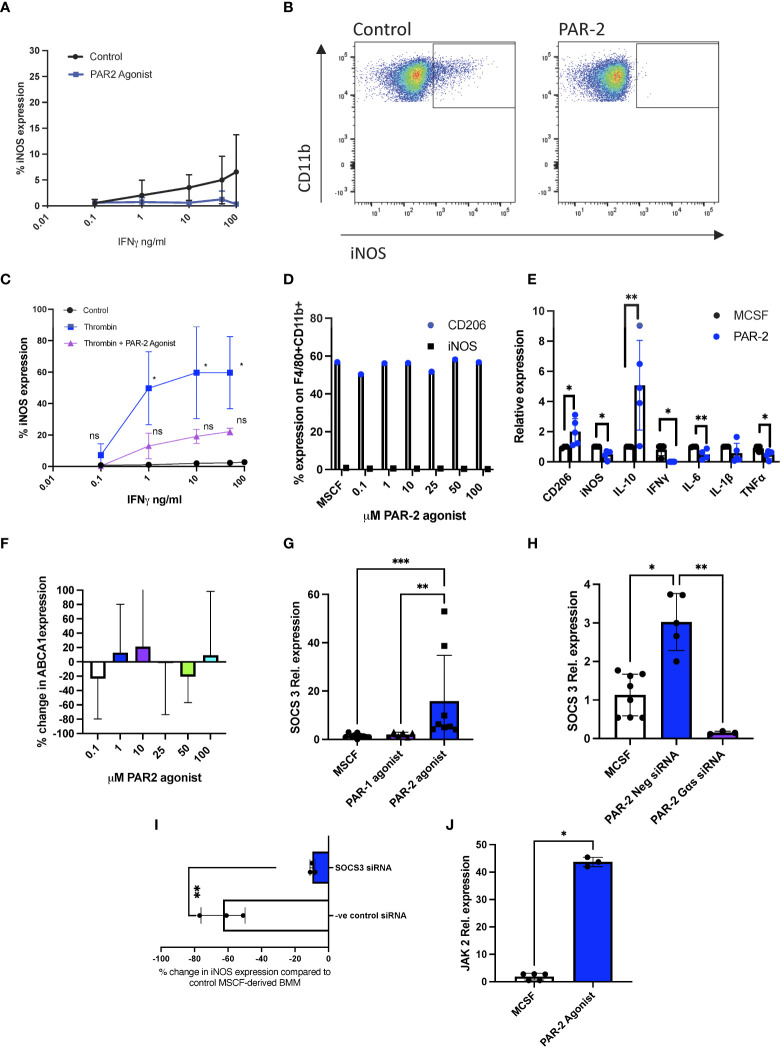
PAR-2 signalling reduces sensitivity to IFNγ by enhancing SOCS3 expression. **(A).** MCSF-derived BMM cultured for 3 hours with 100 µM PAR-2 agonist or maintained in MCSF (control) prior to the addition of escalating concentrations of IFNγ. Cells were then analysed by intracellular flow cytometry for iNOS expression. Data shows results from 3 (PAR-2) and 5 (control) separate groups of experiments. **(B).** Representative flow cytometry profiles of experiment described in Fig1A. **(C).** MCSF-derived BMM incubated with increasing concentrations of IFNγ (control), or primed beforehand with thrombin for 24 hours (Thrombin) or with thrombin and 100 µM PAR-2 agonist prior to IFNγ stimulation. Cells were then analysed by intracellular flow cytometry for iNOS expression. Data shows results from 3 experiments. The significance levels shown are in comparison to MCSF control at the same concentration of IFN. **(D).** CD206 (white bars) or iNOS (black bars) expression on F480+CD11b+ cells analysed by intracellular flow cytometry after 24 hours culture with increasing concentrations of PAR2 agonist. **(E).** qPCR data of MCSF-derived BMM treated for 24 hours with 100μM PAR-2 agonist (n=5) or maintained in MCSF (control) (n=4). **(F).** ABCA1 expression in MCSF-derived BMM incubated for 24 hours with escalating concentrations of PAR-2 agonist before analysis by flow cytometry. Data represents percentage change from control (MCSF) expression. Data from at least 3 separate experiments. **(G, H).** Relative SOCS3 expression by qPCR analysis of MCSF-derived BMM. In F, cells maintained in MCSF (n=10) for 24 hours (circle), or after 24 hours incubation with 100μM PAR-1 agonist (TFLLR-NH2) (n=6) (Triangle) or 100μM PAR-2 agonist (n=8) (Square). In H, cells maintained in MCSF for 24 hours (n=8) (white bar), or after 24 hours incubation with 100μM PAR-2 agonist +/- pre incubation for 24 hours with either 30pmol control siRNA (n=4) (blue bar) or 30pmol siRNA specific for Gαs (n=3) (purple bar). **(I).** MCSF-derived BMM incubated for 24 hours with 30pmol siRNA to SOCS3 (white bar) (n=3) or negative control siRNA (blue bar) (n=3), before a 3-hour incubation with 100µM PAR-2 agonist followed by 24-hour incubation with IFNγ (1ng/ml). iNOS expression compared to baseline and shown as % change in iNOS expression from MCSF treated cells as analysed by flow cytometry. **(J).** Relative JAK2 expression by qPCR analysis of MCSF-derived BMM maintained in MCSF (n=5) (white bar), or after 24 hours incubation with 100μM PAR-2 agonist (n=3) (blue bar). The PAR-2 agonist used in all experiments is 2-Furoyl-LIGRLO-amide. All PCR data expression calculated relative to TBP. Samples were compared using a Mann Whitney U test with two tailed p-values (Fig 1I+J), and Kruskal–Wallis one-way anova for multiple comparisons (Fig1E-H). Bar data represents mean +SD. *P <0.05 **P < 0.01 ***P < 0.001. ns, non significant.

Although there was marked intra-experiment variation, MCSF-derived bone marrow macrophages (BMM) cultured with the PAR-2 agonist 2-Furoyl-LIGRLO-amide appeared to have a blunted response to increasing concentrations of IFNγ, as measured by the upregulation of iNOS, when compared to untreated cells (**Figures 1A, B**). To clarify the effect of the PAR-2 agonist, we first incubated cells with thrombin, which significantly enhanced iNOS expression after exposure to low concentrations of IFNγ, as previously shown (9). This responsiveness was now very obviously blunted by prior incubation with a PAR-2 agonist, which reduced iNOS expression down to levels statistically insignificant from controls (**Figure 1C**). The PAR-2 agonist did not, by itself affect the expression of macrophage mannose receptor (CD206) or iNOS by MCSF-cultured BMM, when analysed by flow cytometry (**Figure 1D**), though RT-PCR analysis indicated significant changes in the steady state levels of mRNA encoding these two molecules (**Figure 1E**), as well as statistically significant changes in mRNA levels of IL-10, IL6, TNFα and IFNγ (**Figure 1E**), consistent with the hypothesis that PAR-2 signalling activated an anti-inflammatory transcriptional program.

In contrast to thrombin-mediated PAR-1 signaling, which impacts IFNγ sensitivity *via* changes in ABCA1 expression (9, 21), incubation with a PAR-2 agonist did not alter ABCA1 expression by MCSF-derived BMM (**Figure 1F**). However, qPCR analysis of the PAR-2 agonist treated cells revealed an increase in steady state levels of an mRNA encoding another negative regulator of IFNγ (22); Suppressor of cytokine signaling 3 (SOCS3) compared to control MCSF-derived BMM (p=0.0291), and in contrast to cells incubated with a PAR-1 agonist (TFLLR-amide), which did not affect SOCS3 mRNA levels (**Figure 1G**). SOCS3 protein expression on monocytes was also shown to be PAR-2 dependent by IHC (data not shown). This increase in SOCS3 mRNA levels was completely inhibited by siRNA against Gαs, the G protein involved in PAR-2-mediated cAMP generation (**Figure 1H**) (23). The change in sensitivity to IFNγ, measured by iNOS expression, induced by the PAR-2 agonist (**Figure 1A**) was abolished by prior incubation of the cells with siRNA against SOCS3, but not by a control siRNA (**Figure 1I**), confirming that SOCS3, induced by PAR-2 signaling *via* Gαs, mediated the negative regulation of IFNγ responsiveness. SOCS3 is induced by JAK2 signalling (24). JAK2 expression, was increased (p=0.0357) in the PAR-2 agonist treated cells (**Figure 1J**).

**Figure 2:**
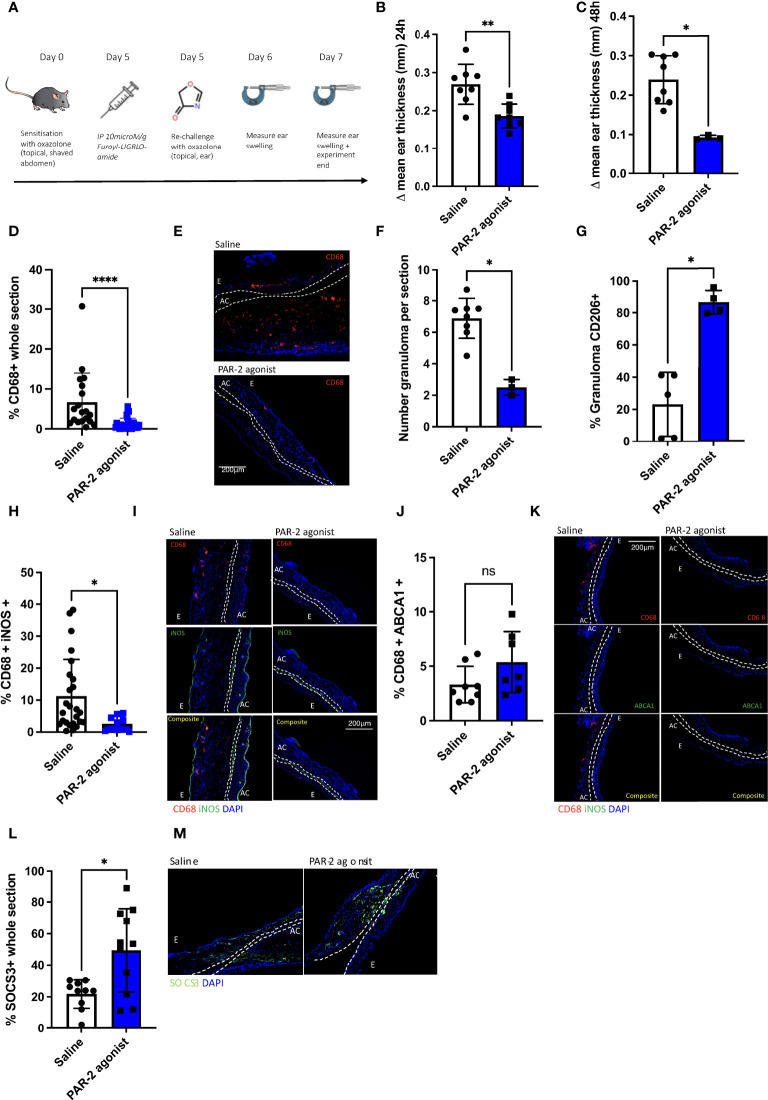
PAR-2 signalling delivers a protective phenotype in oxazolone induced delated type hypersensitivity. **(A).** Schematic illustrating the experimental protocol. C57BL/6 (WT) mice were sensitised on the abdomen with oxazolone on day 0 and re-challenged on day 5 on one of the ears with oxazolone, and on the other ear with vehicle alone. Immediately prior to re-challenge, mice received either IP saline (n= 8) or 10microM/g PAR-2 agonist (n= 7). 24 and 48 hours following re-challenge (days 6 and 7), ear swelling was quantified using a micrometer. Data are presented as Δ mean ear thickness (swelling of oxazolone-treated ear minus that of vehicle treated ear). At least 5 measurements were taken per ear and averaged. **(B, C).** Δ mean ear thickness at 24HRS (day 6) **(B)** and 48HRS (day 7) **(C)** post re-challenge with oxazolone. **(D–M).** Immunofluorescence of frozen sections through oxazolone-treated ears. Bars represent means + SD for saline treated mice (white) or PAR-2 agonist-treated mice (blue). **(D).** Infiltration of CD68^+^ cells: % area of the section occupied by CD68^+^ cells. **(E).** Representative two colour IF sections through oxazolone-painted ears. Images show staining with CD68 (red) and DAPI (blue). **(F).** Number of granuloma per section at 100x magnification: Granuloma was classified as an aggregation of cells co-localisation of DAPI and CD68 at an area of outpouching from the epidermis. **(G).** % CD206 expression within granulomata. **(H–K).** Proportion of CD68^+^ cells co-expressing iNOS (H+I) or ABCA1 (J+K). I+K Representative three colour IF sections through oxazolone-painted ears. Images show staining with CD68 (red) iNOS (green -I), ABCA1 (green -K) and DAPI (blue). **(L, M).** Proportion of whole section SCOS3 expression. Bars represent means + SD for saline treated mice (white) or PAR-2 agonist-treated mice (blue) **(L, M)**. Representative two colour IF sections through oxazolone-painted ears. Images show staining with SOCS3 (green) and DAPI (blue). For representative IF images dotted lines demarcate the auricular cartilage (AC). E= epidermis. All Samples were compared using a Mann Whitney U test with two tailed p-values. Bar data represents mean +SD. *P < 0.05 **P < 0.01 ****P < 0.0001). ns, non significant.

To investigate the role of PAR-2 signalling in a DTH model of oxazolone-induced contact hypersensitivity (25), WT mice were treated intra peritoneally (IP) with the PAR-2 agonist prior to rechallenge with oxazolone (**Figure 2A**). Compared to saline treated controls, the PAR-2 agonist treated mice had reduced ear swelling (ES) at 24 (p= 0.0362) and 48 (p=0.0121) hours (**Figures 2B, C**). Immunohistological examination of the swollen ears at 48 hours showed that PAR-2 agonist-treated mice showed reduced infiltration by CD68^+^ cells compared to the saline-treated control mice (p< 0.0001) (**Figures 2D, E**) and showed a reduction in the average number of granulomas per section (p=0.0021) (**Figure 2F**), whilst at the same time the expression of CD206 (26) within granulomata was significantly increased (**Figure 2G**). In addition, the proportion of CD68^+^ cells expressing iNOS was reduced (p= 0.0017) (**Figures 2H, I)**, whereas the proportion expressing ABCA1 was no different to that seen in control (**Figures 2J, K**). Finally, SOCS3 expression was increased in the ears of mice treated with the PAR-2 agonist (p=0.0399) (**Figures 2L, M**). These data suggests that the SOCS3 expression induced by PAR-2 signalling can impact significantly on the phenotype of DTH responses.

### Basal PAR-2 signaling, regulated by macrophage tissue factor expression, sets the threshold for macrophage sensitivity to IFNγ responsiveness

**Figure 3:**
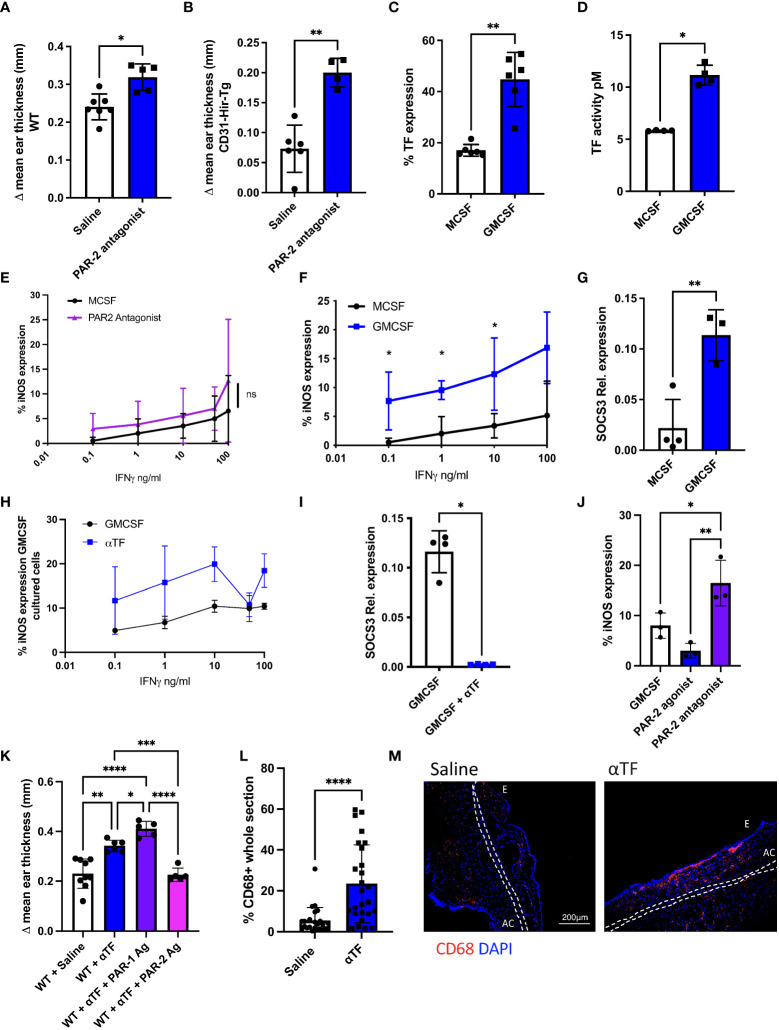
Basal PAR-2 signalling, regulated by macrophage TF expression, sets threshold for macrophage sensitivity to IFNγ responsiveness. **(A, B).** The impact of inhibiting PAR-2 signalling on the outcome of DTH in C57BL/6 (WT-A) or CD31-Hir-Tg mice **a**. Prior to oxazolone re-challenge, mice received IP saline (n= 7 in A; n=6 in B) or 10microM/g PAR-2 antagonist (n= 5 in A; n=4 in B). Data presented as Δ mean ear thickness 24 hours after rechallenge. **(C).** TF expression by day 5 MCSF- or GMCSF-derived BMM, determined by flow cytometry. Data from 6 experiments. **(D).** TF activity of cell lysates from day 5 MCSF (n=4) or GMCSF (n=4)-derived BMM, as measured by production of FXa, when FX provided with FVIIa. **(E).** Day 5 MCSF-derived BMM incubated for 3 hours with MCSF (black circle n=3) or PAR-2 antagonist (purple triangle n=3) before a 24-hour incubation with increasing doses of IFNγ. Data expressed as % of cells expressing iNOS analysed by flow cytometry in presence of PAR-2 antagonist compared to control. **(F).** Day 5 GMCSF-or MCSF-derived BMM incubated for 24 hours with increasing amounts of IFNγ. Data expressed as % of cells expressing iNOS analysed by flow cytometry. Data from 3 experiments. **(G).** Relative SOCS3 expression, assessed by quantitative RT-PCR, by day 5 MCSF (n=4) vs. GMCSF-derived BMM (n=3). **(H).** Day 5 GMCSF-derived BMM incubated for 3 hours with GMCSF or 10mg/ml anti-TF mAb before 24-hour incubation with increasing doses of IFNγ. Data expressed as % of cells expressing iNOS analysed by flow cytometry. Data from at least 3 experiments. **(I).** Day 5 GMCSF-derived BMM incubated with GMCSF (n=4) or 10mg/ml anti-TF-mAb (n=4) for 3 hours before quantitative RT-PCR of SOCS3 expression (relative to TBP). **(J).** Day 5 GMCSF-derived BMM incubated for 3 hours with GMCSF or 100mM PAR-2 agonist or antagonist before a 24-hour incubation with IFNγ (1ng/ml). Data expressed as % of cells expressing iNOS analysed by flow cytometry. Data from 3 separate experiments. **(K).** WT mice were treated with IP saline (n=9) or 10µg/g IP anti-TF Ab (n=6) on day 4 after sensitisation with oxazolone +/- 10µM/g IP PAR-1 agonist (n=5) or PAR-2 agonist (n=5) 24 hours prior to re-challenge with oxazolone. **(L)** Data presented as Δ mean ear thickness. **(L, M)**: IF analysis of CD68^+^ infiltration into the oxazolone-painted ears of mice receiving saline or anti-TF-mAb. **M**: Quantification of CD68^+^ expression. **N**: Representative two colour IF sections through oxazolone-painted ears. Images show staining with CD68 (red) and DAPI (blue). Dotted lines demarcate the auricular cartilage (AC). E= epidermis. Samples were compared using a Mann Whitney U test with two tailed p-values (Fig 3A-D,G,I+L), and Kruskal–Wallis one-way anova for multiple comparisons (Fig3J+K). Bar data represents mean +SD. *P < 0.05 **P < 0.01 ***P < 0.001 ****P < 0.0001. ns, non significant.

In contrast to the effect of the PAR-2 agonist, inhibiting PAR-2 signalling with a PAR-2 antagonist (FSLLRY-amide) prior to oxazolone re-challenge led to increased ES in both WT (**Figure 3A**) and CD31-Hir-Tg mice (**Figure 3B**). Monocytes from this latter strain express the direct thrombin inhibitor hirudin as a membrane fusion protein and are relatively insensitive to IFNγ (9). These data suggest that there is an endogenous, basal PAR-2 signal that acts to limit the extent of the re-call response to oxazolone in both strains.

TF complexed to FVIIa on the surface on the surface of myeloid cells was considered most likely to provide basal signalling through PAR-2 (7). In an early set of experiments, we determined that some peritoneal macrophages could generate their own FVII (**Supplementary Figures 1A, B**), suggesting that mouse TF+ macrophages could generate an endogenous signal through PAR-2. To investigate further, we looked at IFNγ sensitivity in BMM. It was clear that MCSF-derived BMM expressed only low levels TF by flow cytometry (**Figure 3C**) and exhibited a low capacity to generate FXa from FX (**Figure 3D**).

The MCSF-derived BMM were relatively insensitive to IFNγ, as measured by the proportion that expressed iNOS after incubation with increasing concentrations of IFNγ (**Figure 3E**), and prior incubation with a PAR-2 antagonist appeared not to alter this sensitivity. Prior incubation with an inhibitory anti-mouse TF antibody (18) also had little impact on the sensitivity of MCSF-derived BMM to IFNγ (**Supplementary Figure 2A**) but did appear to suppress low basal expression of SOCS3 in these cells (**Supplementary Figure 2B**). Thus, although basal SOCS3 expression in these MCSF-derived BMM appeared to be dependent on TF, they were not otherwise an ideal cell model system to investigate basal PAR-2 signaling.

We therefore generated GMCSF-derived BMM which are reported to express higher TF on the cell surface (27), so to provide a better model to assess TF function. These GMCSF-derived BMM had higher levels of TF by flow cytometry (**Figure 3C**) exhibited higher capacity to generate FXa (**Figure 3D**), were more sensitive to IFNγ than MCSF-derived cells (as assessed by upregulation of iNOS (**Figure 3F**)) and expressed higher basal levels of SOCS3 (**Figure 3G**). On these cells, the inhibitory anti-TF antibody resulted in a trend towards increased sensitivity to IFNγ (**Figure 3H**) and abolished baseline SOCS3 transcription (**Figure 3I**). Additionally, the sensitivity of GMCSF-derived BMM to IFNγ was enhanced by prior incubation with the PAR-2 antagonist (**Figure 3J**) at low concentrations of IFNγ. Finally, sensitivity to IFNγ was reduced by prior incubation with the PAR-2 agonist (**Figure 3J**). These data confirm that basal signalling through PAR-2 suppresses responsiveness to IFNγ (particularly low doses) and support the hypothesis that it is expression of TF (and by virtue its ability to form a signaling complex with FVIIa) that provides this signal through PAR-2, upregulating SOCS3 which acts to suppresses responsiveness to IFNγ.

In the oxazolone-induced contact hypersensitivity model, the inhibitory anti-mouse TF antibody, given IP at 10µg/g 1 day prior to second oxazolone exposure caused an increase in ES at 24 hrs compared to control animals that received saline (p <0.0001) (**Figure 3K**). This was associated with a significant increase in the number of CD68^+^ cells infiltrating the ear (p<0.0001) (**Figures 3L, M**). Administration of the PAR-2 agonist after the anti-TF treatment returned the ES seen to the levels seen in saline-treated mice (**Figure 3K**), in contrast to PAR-1 agonist TFLLR-amide which exacerbated the ES seen after inhibition of TF (p <0.0001) (**Figure 3K**). All these data are consistent with the hypothesis that basal signalling through PAR-2 by TF acts to reduce the sensitivity of BMM to IFNγ and this significantly impacts of the phenotype of DTH responses.

**Figure 4:**
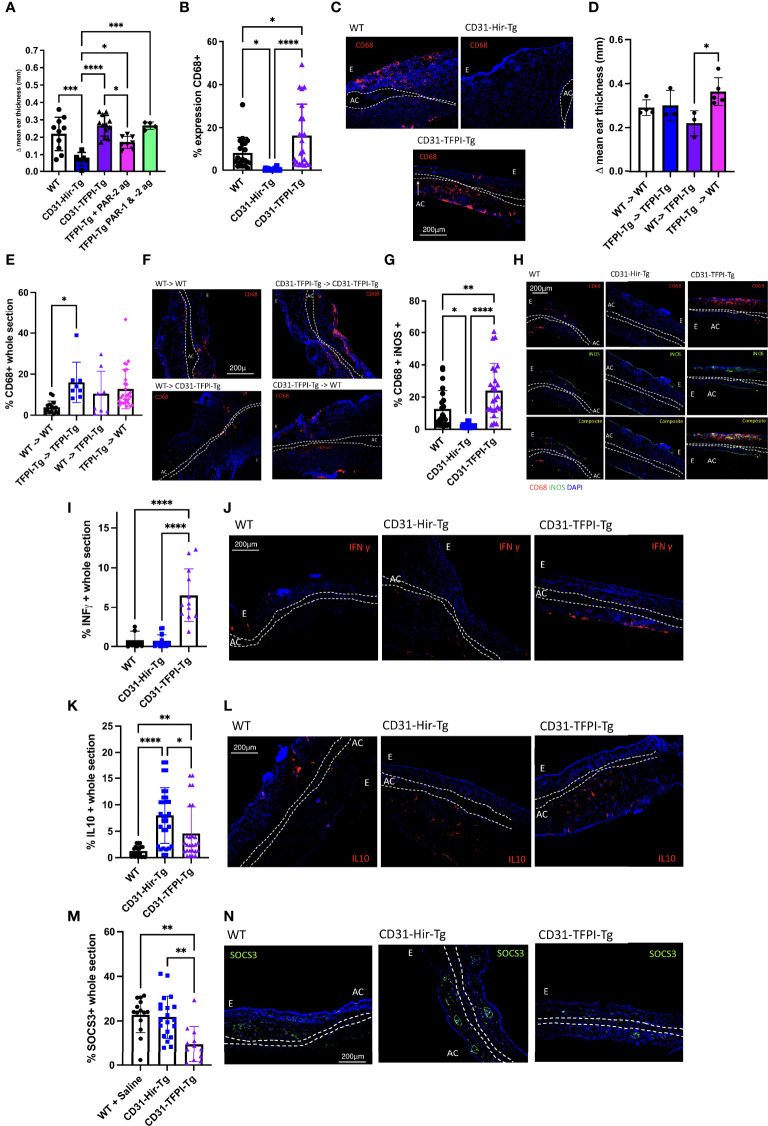
Complete inhibition of TF-mediated PAR signalling on myeloid cells enhances delayed type hypersensitivity responses by removing basal PAR-2 signaling. **(A).** The outcomes of oxazolone induced DTH at 24 hours in C57BL/6 (WT) (white bar n=10), CD31-Hir-Tg (blue bar) (n=6), CD31-TFPI-Tg mice (purple bar) (n=9), CD31-TFPI-Tg mice given 10microM/g PAR-2 agonist (2-Furoyl-LIGRLO-amide) (pink bar) (n=8) and CD31-TFPI-Tg mice given 10microM/g PAR-1 agonist (SFLLR-NH2) and PAR-2 agonist (2-Furoyl-LIGRLO-amide) (green bar) (n=4). The PAR agonists were administered IP immediately prior to oxazolone re-challenge. Data are presented as Δ mean ear thickness. **(B, C).** Immunofluorescence (IF) of frozen sections through oxazolone-treated ears. B. Bars represent means + SD for C57BL/6 (WT) (Black circle), CD31-Hir-Tg (blue square) and CD31-TFPI-Tg mice (purple triangle) for % area of the section occupied by CD68^+^ cells. Experimental groups as above for Fig 4A. **(C).** Representative two colour IF sections through oxazolone-painted ears. Images show staining with CD68 (red) and DAPI (blue). **(D–F).** The outcomes of oxazolone induced DTH in bone marrow (BM) chimeric mice. Experimental groups: WT (CD45.1) BM to (CD45.1) WT (white bar in A, black circle in B) (n=5), CD31-TFPI-Tg BM to CD31-TFPI-Tg (blue bar in A, blue square in B) (n=3), WT (CD45.1) BM to TFPI-Tg (purple bar in A, purple triangle in B) (n=3), TFPI-Tg BM to WT (CD45.1) (pink bar in A, pink diamond in B) (n=6). D. Data are presented as Δ mean ear thickness 24 hours after rechallenging with oxazolone. E. IF of frozen sections through oxazolone-treated ears. Bars represent means + SD % of area of the section occupied by CD68^+^ cells. F. Representative two colour IF sections through oxazolone-painted ears. Images show staining with CD68 (red) and DAPI (blue). **(G–N)** IF of frozen sections through oxazolone-treated ears of WT (black circle), CD31-Hir-Tg (blue square) & CD31-TFPI-Tg mice (purple triangle), showing % of CD68^+^ cells co-expressing iNOS **(G)**. **(H).** Representative three colour IF sections through oxazolone-painted ears. Images show staining with CD68 (red) iNOS (green) and DAPI (blue) (experimental groups as above for Fig 4A). **(I–N)**: % area of the section occupied by IFNγ **(I)**, IL-10 **(K)** or SOCS3 **(M)**. **(J, L, M)** Representative two colour IF sections through oxazolone-painted ears. Images show staining with IFNγ (red -J), IL-10 (red – L), SOCS3 (green- N) and DAPI (blue) (n numbers as 4A). For IF least 3 sections per mouse were analysed. For ES data at least 5 measurements were taken per ear and the results averaged. Dotted lines demarcate the auricular cartilage (AC). E= epidermis. All samples were compared using Kruskal–Wallis one-way anova for multiple comparisons Bar data represents mean +SD. *P < 0.05 **P < 0.01 ***P < 0.001 ****P < 0.0001.

To link these findings in the oxazolone-induced hypersensitivity model to TF and PAR-2 signalling on myeloid cells, we used CD31-TFPI-Tg mice. This strain of mice expresses human tissue factor pathway inhibitor (TFPI) covalently linked to the membrane proximal domain of human CD4, as a transgenic fusion protein on all CD31+ cells including monocytes (28). The TFPI binds TF, FVIIa and FXa and inhibits FXa generation from FX (29) thus inhibiting TF-dependent thrombin generation (28), but will also inhibit basal TF signaling through PAR-2. Compared to CD31-Hir-Tg mice, which develop a reduced response in the DTH model *via* a mechanism we have previously defined (9), the CD31-TFPI-Tg mice had exaggerated ES at 24 (**Figure 4A**) and 48 (**Supplementary Figure 3**) hours after second oxazolone exposure. Although compared to WT, CD31-TFPI-Tg mice had similar degrees of ES at either time point (**Figure 4A** & **Supplementary Figure 3**), they did show increased infiltration of CD68^+^ cells compared to both WT and CD31-Hir-Tg (**Figures 4B, C**). The degree of ES reduced significantly when the CD31-TFPI-Tg mice were treated with the PAR-2 agonist prior to rechallenge with oxazolone (p=0.0495) (**Figure 4A**), but this effect was abolished by co-administration of the PAR-1 agonist TFLLR-amide (**Figure 4A**). BM reconstitution experiments confirmed that these changes in response to second oxazolone challenge were dependent on expression of the TFPI transgene by BM-derived cells (**Figures 4D–F**). Compared to WT and CD31-Hir-Tg mice, more of the CD68^+^ cells infiltrating the ear co-stained for iNOS (**Figures 4G, H**) and fewer co-expressed CD206 (**Supplementary Figures 4A, B**). Finally, the ears contained more cells expressing IFNγ (**Figures 4I, J**), but fewer expressing IL-10 (**Figures 4K, L**) or SOCS3 (**Figures 4M, N**).

All these data support the hypothesis that TF expression on myeloid cells initiates a basal PAR-2 signal that acts to enhance SOCS3 expression, which, during a DTH response limits ear swelling, inhibits infiltration by CD68^+^ cells and diminishes the expression of iNOS and whilst enhancing the expression of CD206 and IL-10.

### Combining PAR-2 agonist with PAR-1 antagonist activity

**Figure 5:**
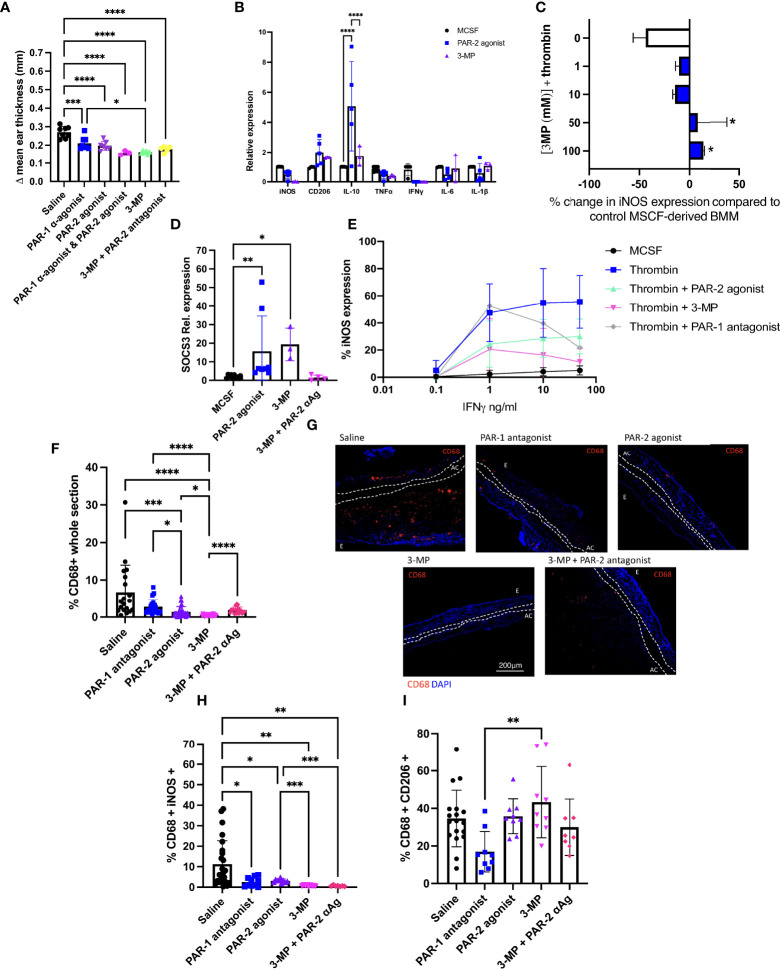
3-MP inhibits IFNγ signalling through both PAR-1 antagonism and PAR-2 agonism. **(A)** The impact of combination PAR-1 antagonism and PAR-2 agonism on the outcome of Ox-DTH. Immediately prior to re-challenge with oxazolone, C57BL/6 (WT) mice received either IP saline (n= 8) or 10microM/g IP FLLRN (PAR-1 antagonist (α-agonist) (n=8), 2-Furoyl-LIGRLO-amide (PAR- 2 agonist) (n=7), FLLRN combined with 2-Furoyl-LIGRLO-amide (n=3), 3-MP (n=6) or 3-MP + FSLLRY-Amide (PAR-2 antagonist) (n=5). Data represented as Δ mean ear thickness. **(B)** qPCR data for day 5 MCSF-derived BMM incubated for 24 hours with 100μM PAR-2 agonist or 100μM 3- MP or maintained in MCSF alone. Expression calculated relative to TBP. Data from 3 separate experiments. **(C)** Day 5 MCSF-derived BMM were cultured for 2 hours with escalating concentrations of 3-MP prior to thrombin stimulation for 24 hours. Control cells were incubated in thrombin alone (white bar). Cells were then analysed by flow cytometry for surface ABCA1 expression. Data from 3 separate experiments. Significance compared to control conditions (white bar). **(D).** qPCR for SOCS3 expression by control MCSF-derived BMM maintained in MCSF for 24 hours (black circle) (n=10), or after 24 hours incubation with 100μM PAR-2 agonist (blue square) (n=8) or 3-MP (purple triangle) (n=3) with or without a 2-hour pre incubation with PAR-2 antagonist (αAg) (FSLLRY-NH2) (pink diamond) (n=4). Expression calculated relative to TBP. **(E)** MCSF-derived BMM incubated with increasing concentrations of IFNγ alone (=‘MCSF’), or primed with thrombin for 24 hours (Thrombin) or with thrombin and 100 µM 2-Furoyl-LIGRLO-amide (Thrombin +PAR-2 agonist), 3-MP (Thrombin + 3-MP) or FLLRN (Thrombin + PAR-1 antagonist), prior to IFNv stimulation (thrombin and PAR-2 agonist remained in culture). Cells were then analysed by intracellular flow cytometry for iNOS expression. Data shows results from 3 experiments. **(F–I)** Experimental groups and n numbers as described in Figure 5A. Immunofluorescence of frozen sections through oxazolone-treated ears. Bars represent means + SD for saline treated mice (white), IP FLLRN (PAR-1 antagonist) (blue), 2-Furoyl-LIGRLO-amide (PAR- 2 agonist) (purple), 3-MP (pink) or 3-MP and FSLLRY-NH2 (3-MP + PAR-2 antagonist- αAg) (red). **(F)** % area of the section occupied by CD68^+^ cells. **(G)** Representative two colour IF sections through oxazolone-painted ears. Images show staining with CD68 (red) and DAPI (blue). **(H–I)**: % of CD68^+^ cells co-expressing iNOS **(H)** or CD206 **(I)**. For ES data at least 5 measurements were taken per ear and the results averaged. Dotted lines demarcate the auricular cartilage (AC). E= epidermis. Samples were compared using Kruskal–Wallis one-way anova for multiple comparisons (Fig 5A,C,D,F,H+I) or two way anova (Fig5B) Bar data represents mean +SD. * P< 0.05 **P < 0.01 ***P < 0.001 **** P < 0.0001.

We have previously demonstrated that inhibiting thrombin signaling through PAR-1 on the surface of myeloid cells either by transgenic expression of hirudin or by injection of a cell membrane localising thrombin inhibitor (PTL060), acts to diminish the recall DTH response to oxazolone (9). In the same model, IP treatment with a PAR-1 antagonist 1 day prior to second oxazolone exposure reduced ES significantly (**Figure 5A**), as expected, as did a PAR-2 agonist (**Figure 5A**). A combination of both the PAR-1 antagonist and PAR-2 agonist caused a reduction in ES that was greater than either agent alone (p < 0.001) (**Figure 5A**), suggesting that the effects were additive and consistent with different pathways being influenced.

3-MP is a PAR-1 antagonist that has been reported to have agonistic properties for PAR-2 (30). We confirmed this dual action in an *in vitro* chemokine secretion assay (see Supplementary Figure 5 and supplementary text). Furthermore, MCSF-derived BMM incubated with 3-MP showed similar patterns of expression of inflammatory markers to those induced by the selective PAR-2 agonist with a significant increase in IL-10 expression (p= 0.0179) compared to control MCSF treated cells (**Figure 5B**). Treatment of MCSF-derived BMM with 3-MP prevented thrombin-mediated down regulation of ABCA1 (PAR-1 antagonist component) (**Figure 5C**) but enhanced SOCS3 expression (p=0.0124) equivalent to that induced by the selective PAR-2 agonist (**Figure 5D**). The increase in SOCS3 expression induced by 3-MP was inhibited by pre-treating the MCSF-derived BMM with the PAR-2 antagonist for 2 hours prior to 3-MP stimulation (**Figure 5D**). Finally, 3-MP dramatically reduced the thrombin-mediated heightened sensitivity of MCSF-derived BMM to IFNγ (**Figure 5E**), with an impact that appeared to correlate entirely with the combined changes induced separately by the PAR-2 agonist and PAR-1 antagonist (**Figure 5E**). All these data further support the conclusion that 3-MP has simultaneous PAR-1 antagonist and PAR-2 agonist properties.

In the DTH model, 3-MP led to a reduction in ES that was equivalent to the combination of individual reagents (**Figure 5A**). Mice treated with 3-MP had minimal CD68 infiltration into thek ears (**Figures 5F, G**), with fewer of these cells expressing iNOS (**Figure 5H**; **Supplementary Figure 6A**) and more expressing CD206 (**Figure 5I**; **Supplementary Figure 6B**). Co-administration of 3-MP with a PAR-2 antagonist led to a non-significant increase in ES (p=0.0823) (**Figure 5A**) but did significantly increase CD68^+^ cell infiltration (**Figures 5F, G**) and partially reversed the proportion of these cells expressing either CD206 (**Figure 5I**; **Supplementary Figure 6A**). These data are entirely consistent with the notion that provision of a PAR-2 signal enhances, through a different mechanism, the anti-inflammatory impact of inhibiting PAR-1 signalling.

### ‘Cytotopic’ modification enhances the biological activity of 3-MP in the DTH response

3-MP was modified into the ‘cytotopic’ peptide PTL0GC-1 by conjugating with a synthetic myristoyl switch, and activity compared to 3-MP in the oxazolone-induced contact hypersensitivity model. As a control for these experiments, we tried to generate a ‘cytotopic’ peptide from the selective PAR-1 antagonist FLLRN but attempts to modify the sequence for the conjugation reaction resulted in either loss of solubility or loss of PAR-1 antagonistic activity (data not shown). We therefore compared PT0GC-1 to the cytotopic direct thrombin inhibitor PTL060.

**Figure 6:**
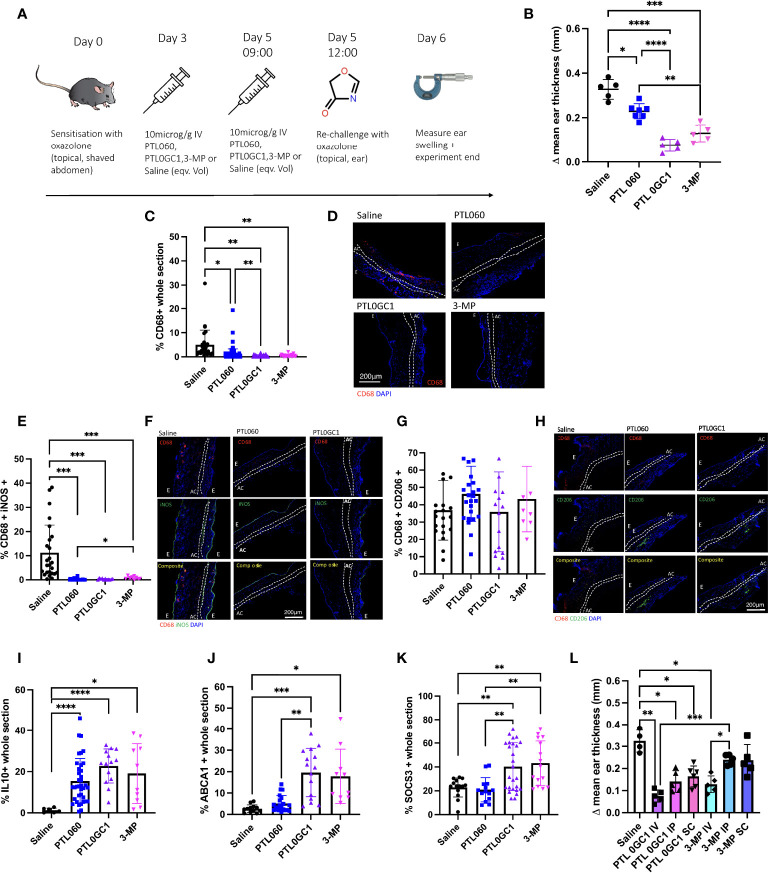
PTL0GC-1 provides a protective benefit in the outcome of type IV hypersensitivity greater than PTL060. **(A)** Schematic illustrating the experimental protocol. C57BL/6 (WT) mice were sensitised on the abdomen with oxazolone on day 0 and re-challenged on day 5 on one of the ears with oxazolone, and on the other ear with vehicle alone. On day 3 and 5 mice received either IV saline (n=5), or 10microM/g IV PTL060 (n=7) or equimolar PTL0GC-1 (n=5) or 3-MP (n=5). 3 hours after injection on day 5 mice were then re-challenged with oxazolone (on the right) or vehicle alone (on the left). **(B)** Results are expressed as change in mean ear thickness between treated (right ear) and untreated (left ear) at 24 hours. At least 5 measurements were taken per ear and the results averaged. **(C–L)** Immunofluorescence of frozen sections through oxazolone-treated ears (groups as described in Fig 6A). Bars represent means + SD for saline (black circle), PTL060 (blue square), PTL0GC-1 (purple triangle) or 3-MP (pink diamond) treated mice. **(C)** % area of the section occupied by CD68^+^ cells. **D.** Representative two colour IF sections through oxazolone-painted ears. Images show staining with CD68 (red) and DAPI (blue). **(E–H)** % of CD68^+^ cells co-expressing iNOS **(E)**, CD206 **(G)**. Representative three colour IF sections through oxazolone-painted ears. Images show staining with CD68 (red) iNOS (green - F)/CD206 (green - H) and DAPI (blue). **(I–K)** % area of the section occupied by IL10 **(I)**, ABCA1 **(J)** or SOCS3 **(K)**. At least 3 sections per mouse were analysed. **(L)** Evaluating other routes of delivery of PTL0GC-1 or 3-MP. On day 3 and 5 oxazolone primed mice were treated with saline (n=4) or 7.5 µg/g PTL0GC-1 IV (n=5), IP (n=5), SC (at double dose) (n=6) or 3-MP or IV (n=5), IP (n=5) or SC (n=5) 3-MP (at the equivalent molarity of PTL0GC-1). 3 hours after injection on day 5 mice were then re-challenged with oxazolone (on the right) or vehicle alone (on the left). Results are expressed as change in mean ear thickness between treated (right ear) and untreated (left ear) at 24 hours. For ES data at least 5 measurements were taken per ear and the results averaged. Dotted lines demarcate the auricular cartilage (AC). E= epidermis. All samples where compared using Kruskal–Wallis one-way anova for multiple comparisons. Bar data represents mean +SD. *P <0.05 **P < 0.01 *** P < 0.001 ****P < 0.0001.

WT mice treated with IV 7.5 µg/g PTL0GC-1 on day 3 and 5 prior to second rechallenge with oxazolone (**Figure 6A**) had less ES than mouse controls treated with either saline or equimolar PTL060 or 3-MP (**Figure 6B**). Examination of the ears by immunofluorescence showed that the inflamed ears of PTL0GC-1-treated mice contained significantly reduced numbers of CD68^+^ cells (**Figures 6C, D**) vs. all control conditions. There was significantly decreased expression of iNOS in CD68^+^ cells in all treatments relative to saline treated controls (**Figures 6E, F**). CD206 expression remained unchanged across all groups (**Figures 6G, H**). PTL0GC-1 induced the greatest expression of IL-10 (**Figure 6I**; **Supplementary Figure 7A**) and ABCA1 (**Figure 6J**; **Supplementary Figure 7B**) of the three active compounds, and only treatment with PTL0GC-1 or 3-MP induced increased expression of SOCS3 (**Figure 6K**; **Supplementary Figure 7C**). IV treatment with 3-MP was more effective at reducing ES than either IP or S/C administration, but for each of these routes of administration, equimolar concentrations of PTL0GC-1 resulted in superior reduction in ear swelling (**Figure 6L**). These data indicate that ‘cytotopic’ modification of 3-MP endows superior anti-inflammatory activity, compared to the parental 3-MP and compared to cytotopic inhibition of thrombin alone.

## Discussion

In the work presented here we show that signalling through PAR-2 reduces myeloid cell sensitivity to IFNγ *via* upregulation of the well-described IFNγ signaling inhibitor SOCS3. This can be exploited to reduce the swelling and myeloid cell infiltrate associated with the murine DTH response to the skin sensitizer oxazolone. Moreover, there is a basal signal delivered through PAR-2 by macrophage-expressed TF, that serves to dampen endogenous sensitivity to IFNγ, which when interrupted enhances the DTH response. Having previously defined the importance of PAR-1 signaling for murine DTH responses (9), we now show that combining PAR -1 antagonism with PAR-2 agonism has an additive inhibitory effect on development of DTH, and that both activities are combined in the molecule 3-MP, which therefore has more potent effect than when either agent is administered alone. Finally, we show that addition of a cell membrane localizing tail to 3-MP, to generate a novel compound PTL0GC1 further enhances biological activity and leads to near total suppression of the DTH response after oxazolone.

Using a combination of MCSF- and GMCSF-derived BMM, that differed significantly in the amount of TF expressed, we demonstrated that a PAR-2 agonist induced an anti-inflammatory mRNA pattern without inducing significant phenotypic changes such as polarisation. This resulted in significant attenuation of both basal and thrombin-primed responsiveness to IFNγ measured by how much iNOS expression was changed by increasing concentrations of IFNγ. The dampened responses to IFNγ was completely inhibited by siRNA targeting of Gas and SOCS3, indicating that the mechanism involved cAMP dependent upregulation of SOCS3, which is a known regulator of IFNγ signal transduction (31). In contrast, a PAR-2 antagonist significantly enhanced iNOS expression induced by low concentrations of IFNγ, but only on GM-CSF-derived BMM which expressed detectable amounts of TF and had the ability to generate FXa from FX, suggesting there was a basal signal through PAR-2, associated with TF expression. Basal SOCS3 expression in both types of BMM was abolished by incubation with an inhibitory antibody against mouse TF, which also significantly enhanced basal response to low concentrations of IFNγ in GMCSF-derived BMM. These data strongly suggest that TF expressed on the surface of BMM was signalling through PAR-2 to promote basal SOCS3 expression. We have not investigated the downstream signaling pathways from SOCS3 in this system which we acknowledge is a weakness in this work. In addition, we have only addressed IFNγ signalling in this model, as it is the key mediator of the type 4 hypersensitivity response in sterile inflammation (25). We propose that TF acts alongside IFNγ to increase iNOS expression through PAR-1 in a manner similar to pathogen-derived TLR4 signalling does in non sterile inflammation (32). We have not systematically studied whether the PAR-2 signalling consequences of TF also act to modulate TLR-mediated iNOS expression, which we acknowledge is another weakness in our work.

*In vivo* these findings were mirrored in a model of oxazolone-induced DTH where a PAR-2 agonist given to mice prior to re-challenge with oxazolone resulted in reduced ear swelling and the reduced number of CD68^+^ cells infiltrating the lesion adopted an anti-inflammatory phenotype associated with increased SOCS3 expression. The reverse was true for a PAR-2 antagonist, which led to increased ear swelling. In addition, an inhibitory anti-mouse TF antibody given to the mice prior to re-challenge with oxazolone caused a significant increase in ear swelling, and the ears showed enhanced infiltration by CD68^+^ cells. This effect was completely reversed by simultaneous administration of a PAR-2 agonist. All these data are consistent with the hypothesis that signalling through PAR-2, induced by TF, drives upregulation of SOCS3, which inhibits the sensitivity of monocytes/macrophages to IFNγ *in vivo* and profoundly influences the phenotype of DTH responses.

SOCS3 has previously been studied in both DTH and atherosclerosis. In DTH, SOCS3 expression by CD206+ cells were associated with reduced ear swelling (33), and SOCS3-deficient mice showed exaggerated responses, consistent with the notion that SOCS3 dampens responsiveness of macrophages to inflammatory stimuli. In atherosclerosis, antisense suppression of SOCS3 in APOE -/- mice exacerbated atheroma development (34), whereas adenovirus-mediated upregulation of SOCS3 in the same model suppressed plaque development, in association with reduction in STAT1 and STAT3-dependent gene expression (35). Most recently, increased expression of SOCS3 specifically in plaque macrophages following specific deletion of a repressor, EZH2 (36), significantly reduced atheroma development (37). All these data are consistent with the data presented here. Although we have not addressed the role of EZH2 in our system, to the best of our knowledge, ours is the first to link PAR-2 signalling induced by TF on myeloid cells to basal expression of SOCS3.

Coagulation proteases have an elegant relationship with inflammation. Thrombin, the ultimate serine protease generated by the coagulation cascade after TF activation is able to prime innate immune cells to bestow heightened sensitivity to proinflammatory signalling (9, 14). In the data presented here we demonstrate a different consequence of TF signalling – a phenotype of reduced sensitivity to IFNγ mediated *via* PAR-2. We hypothesise that the balance between these two opposing actions of TF controlled will be controlled *in vivo* by the encryption status of the TF expressed on myeloid lineage cells.

Several mechanisms have been implicated in the post-translational control of TF “encryption” and “decryption”. At resting state, TF is encrypted with very little coagulant activity as evidenced by its low affinity for FVIIa (K_d_ of 5-20nM) (38). Decrypted TF is procoagulant and has been shown to rapidly bind FVIIa (K_d_ <1nM) and is able to effectively cleave FX (39). The process by which TF is encrypted and decrypted remains poorly defined and is a source of some controversy. A number of hypotheses have been proposed for the maintenance of encrypted TF such as: presence of unpaired cysteine thiols at Cys-186 and Cys-209 (40), a neutral phospholipid environment (41), cell surface dimerization (42) and sequestering of TF in lipid rafts (43). It is possible that all of these mechanisms contribute to some extent but there remains no consensus on which is the dominant mechanism. However it is certain that encrypted TF can mediate PAR-2 signaling; Pendurthi et al. demonstrated that TF : FVIIa induced PAR-2 signaling in the absence of the Cys186-Cys209 disulphide bond (7) which is critical for activation of FX (44). Our demonstration that a proportion of resting murine macrophages can make FVII suggests that some TF-expressing myeloid cells have all they need to maintain a basal PAR-2 signal.

In light of our previous dissection of the importance of thrombin-mediated PAR-1 signalling on myeloid cells for DTH and other pro-inflammatory responses (9, 10), our data suggests that this basal PAR-2 signal dampens these responses and limits the inflammation that results. With this in mind, we tested the activity of 3-MP, which has been widely used experimentally as a PAR-1 antagonist (45–47), but it is long been recognised that it has a dual action, acting as an agonist at PAR-2, whilst inhibiting PAR-1 (30). MCSF-derived BMM incubated with 3-MP activated the same anti-inflammatory programme as that induced by a selective PAR-2 agonist, increased expression of SOCS3 and additionally prevented thrombin-mediated downregulation of ABCA1. Thus, 3-MP was superior to either a selective PAR-1 antagonist or PAR-2 agonist at dampening the enhanced sensitivity to IFNγ induced by thrombin. This translated *in vivo* in the DTH model, such that 3-MP treated mice had minimal ear swelling and macrophage infiltration after second exposure to oxazolone.

Therapeutically manipulating coagulation proteases to limit the inflammatory responses induced by thrombin through PAR-1 has been fraught with problems induced by the necessary impact on haemostasis, which means that otherwise promising compounds have limited use due to their unwanted side effects (48). This applies also to compounds that antagonise PAR-1 without inhibiting thrombin (49). We previously showed that tethering a myristoyl electrostatic switch to the direct thrombin inhibitor hirulog, to make PTL060, allowed therapeutic uncoupling of these unwanted anticoagulant effects of from the anti-inflammatory effect of inhibiting PAR-1 signalling (10). This was our rationale for chemically modifying 3-MP to make PTL0GC-1. As expected, because of the PAR-2 agonist activity, PTL0GC-1 had superior biological activity compared to PTL060. However, surprisingly, a single dose was also better than an equimolar dose of 3-MP in the oxazolone DTH model, when delivered IV, IM or s/c. Although we have not directly investigated why, we speculate it relates to the targeting of the molecule to lipid membranes. We have previously demonstrated that the cytotopic tail localises the compound to cell membrane where it remains active for a longer period than the untailed compound (10). Thus, membrane targeting keeps 3-MP in the vicinity of the target receptor for a prolonged period.

These new data are inconsistent with published evidence that PAR-1 and PAR-2 have similar pro-inflammatory functions and that signalling through each induces similar effects. In DTH models, Kawagoe et al. (50) showed that PAR-2 deficient mice showed reduced ear swelling compared to controls, and Barr et al. (51) used the PAR-2 antagonist PZ-235 to reduce ear swelling after DTH in WT mice. Although this new data is entirely consistent with previous work we’ve reported on the role that TF and PAR-2 play in the ability of murine dendritic cells to prime T cells (16), it is very different to previous data we’ve reported before in different models using CD31-TFPI-Tg mice and CD31-Hir-Tg mice. In all our other studies to date, TFPI and hirudin expression has had the same functional impact. In contemplating why these new data may conflict with what’s been reported before, our hypothesis is that a combination of two factors is relevant. First, this appears to be a myeloid cell-specific phenotype. Second, PAR-2 signalling is acting to prime cells before exposure to a second stimulus. There are only a few other examples in the literature where PAR-2 agonists have been used to ‘prime’ myeloid cells, prior to exposure to a second stimulus. Gleeson et al. (27) used GMCSF-derived BMM (from BALB/c mice) and primed with FXa before stimulation with LPS. It led to significant inhibition of LPS-induced TNFα and IL-6 production, which was PAR-2 dependent. Similarly, Steven et al. (52), using human monocyte GMCSF-derived macrophages showed that PAR-2 agonist stimulation reduced LPS-induced TNFα production, whereas Garcia-Gonzalez et al. (53) showed that PAR-2 priming inhibited subsequent MTB CFP-induced IL-12 and IFNγ production by human monocytes. Although in both the latter reports, the effect of PAR stimulation was complex, being both context dependent in the former (i.e., only occurring in GMCSF-derived macrophages) and promoting an M2 phenotype in the latter, nevertheless, all these data are consistent with our data that PAR-2 priming significantly reduces the subsequent responsiveness of macrophages to pro-inflammatory stimuli. We have not addressed the impact of PAR-2 signalling on human monoctyes which is a weakness in this paper.

In summary, in combination with the detailed work we have previously presented in the DTH model our novel data support the hypothesis that TF on myeloid cells initiates coagulation protease activation, the products of which are capable of signalling through both PAR-2 and simultaneously through PAR-1. The former upregulates SOCS3 expression and inhibits signal transduction through the IFNγ receptor (and dampens responsiveness to IFNγ), whilst the latter enhances responsiveness to IFNγ, *via* degradation of ABCA1 and movement of cell membrane IFNγ receptor into cholesterol-rich microdomains. These two competing effects provide a ‘priming’ mechanism capable of modifying responses of myeloid cells to inflammatory stimuli. This understanding allowed us to develop a novel therapeutic, PTL0GC-1, capable of combined inhibition of PAR-1 with activation of PAR-2, which we have demonstrated provides significant suppression of DTH responses when administered *via* various different routes.

## Data availability statement

The raw data supporting the conclusions of this article will be made available by the authors, without undue reservation.

## Ethics statement

The animal study was reviewed and approved by Kings College London LREC.

## Author contributions

HW designed and performed all the experiments and wrote the manuscript. AD designed the experiments, supervised the overall project and assisted in manuscript preparation. HL and DC assisted with *in vitro* experimentation. RS developed the cytotopic compounds used in this project. DK supplied the anti-TF antibodies used in this work. MR and JM assisted in experimental design and manuscript review. All authors contributed to the article and approved the submitted version.

## Funding

Support for this work was received through an MRC research training fellow grant MR/P018513/1 and MRC Confidence in Concept MC_PC_18052. KCL have awarded or submitted patents covering therapeutic use of PTL060 and PTL0GC-1.

## Acknowledgments

We acknowledge the contribution of Michele Weber, Fonds National de la Recherche Luxembourg, who performed the initial experiments that initiated this work.

## Conflict of interest

DK is employed by Genentech, Inc.

The remaining authors declare that the research was conducted in the absence of any commercial or financial relationships that could be construed as a potential conflict of interest.

## Publisher’s note

All claims expressed in this article are solely those of the authors and do not necessarily represent those of their affiliated organizations, or those of the publisher, the editors and the reviewers. Any product that may be evaluated in this article, or claim that may be made by its manufacturer, is not guaranteed or endorsed by the publisher.
