# Supplementary material for: Manipulation of tissue factor-mediated basal PAR-2 signalling on macrophages determines sensitivity for IFNγ responsiveness and significantly modifies the phenotype of murine DTH
Source: Front Immunol. 2022 Sep 12;13:999871. doi: 10.3389/fimmu.2022.999871 (PMC9510775; doi:10.3389/fimmu.2022.999871)
Supplement: Supplementary file 1 [file DataSheet_1.docx]

**Supplementary Text**

**In vitro chemokine assay**

We used a previously described in vitro chemokine secretion assay (1) to assess whether 3-MP behaved differently to the selective PAR-1 antagonist SFLLRN-Amide. First, we showed that both MIF and CCL2 were secreted after incubation with the PAR-1 agonist SFLLRN-Amide, but less so after incubation with either the PAR-2 agonist or 3-MP (**SF** 5A). In contrast, CX3CL1 and CCL5 were secreted at equal levels after exposure to all three agents. These data indicate that in these assays, 3-MP behaves like a PAR-2 agonist. In assays where thrombin was used as the PAR-1 activator to stimulate chemokine secretion, the expression of MIF and CCL-2 was reduced by the selective PAR-1 antagonist (SFLLRN-Amide) and also by 3-MP, but not by either the PAR-2 antagonist FSLLRY-Amide or the PAR-2 agonist, indicating that in these assays, 3-MP behaved like a PAR-1 antagonist (**SF** 5B). In contrast, the thrombin-dependent secretion of CCL-5 (and to some extent CX3CL1) was partially inhibited by the PAR-1 antagonist SFLLRN-Amide (**SF** 5B) but enhanced by the PAR-2 agonist: 3-MP had a neutral impact on the thrombin-dependent secretion of either chemokine.^[[1]](#footnote-1)^ These data fully support the conclusion that 3-MP behaves simultaneously as a functional PAR-1 antagonist and PAR-2 agonist.

**Supplementary Figures**

**Supplementary Figure 1.**

**Supplementary Figure 1: FVII expression on PM**

A. qPCR data of FVII expression on WT peritoneal macrophages. Expression calculated relative to TBP.

B. Immunofluorescence analysis of CD45 and FVII expression on WT peritoneal macrophages.

**Images relate to Figure 3**

**Supplementary Figure 2.**

A

B

ns

**Supplementary Figure 2: Response of MCSF cultured BMM to anti -TF mAb**

A. MCSF-derived BMM incubated for 3 hours with MCSF (black line) or 10mg/ml anti-TF mAb (G-blue line) before a 24-hour incubation with increasing doses of IFNγ. Data expressed as % of cells expressing iNOS analysed by flow cytometry.

B MCSF-derived BMM incubated with MCSF (n=4) or 10mg/ml anti-TF-mAb (n=3) for 3 hours before quantitative RT-PCR of lysates to assess SOCS3 expression (relative to TBP).

**Images relate to Figure 3**

**Supplementary Figure 3.**

**Supplementary Figure 3: Outcome of oxazolone induced DTH in transgenic mice at 48 hours**

The outcomes of oxazolone induced DTH at 48 hours in C57BL/6 (WT) (white bar n=10), CD31-Hir-Tg (blue bar) (n=6) and CD31-TFPI-Tg mice (purple bar) (n=7). Data are presented as Δ mean ear thickness.

**Image relates to Figure 4**

**Supplementary figure 4**

A

B

**Supplementary Figure 4: CD206 expression in transgenic mice in response to oxazolone**

A. IF of frozen sections through oxazolone-treated ears of 3 of WT, CD31-Hir-Tg & CD31-TFPI-Tg mice, showing % of CD68+ cells co-expressing CD206

B. Representative three colour IF sections through oxazolone-painted ears. Images show staining with CD68 (red) CD206 (green) and DAPI (blue). Dotted lines demarcate the auricular cartilage. AC = auricular cartilage, E= epidermis.

**Image relates to Figure 4**

**Supplementary Figure 5.**

[chemokine] (pg/ml)

0.0001

0.00001

0.0001

0.0001

0.24

0.36

0.26

0.04

B

A

[chemokine] (pg/ml)

0.008

0.6

0.9

0.0002

0.03

0.5

0.5

0.04

0.09

0.3

0.06

0.44

0.003

0.01

0.02

0.4

**Supplementary Figure 5: Chemokine expression by Mouse SMC in response to 3-MP**

A+B. Chemokine concentration analysed by ELISA in the supernatants of primary mouse smooth muscle cells (MSMCs). In A, cells serum starved for 24hours then incubated for 12 hours with 10 µM H-Ser-Phe-Leu-Leu-Arg-Asn-NH2 (SFLLRN-Amide) (PAR1 Ag) (black bars), 2-Furoyl-LIGRLO-Amide (PAR2 Ag) (white bars) or 3-MP (grey bars), before the medium was changed, and supernatants harvested 48 hours later.

In B, after serum starving for 24 hours, cells incubated for 12 hours with 10mM H-Phe-Leu-Leu-Arg-Asn-OH (FLLRN) (PAR-1 antag) (upsloping hatches), H-Phe-Ser-Leu-leu-Arg-Tyr-NH2 (FSLLRY-Amide) (PAR-2 antag) (down sloping hatches), 2-Furoyl-LIGRLO-Amide (PAR2 Ag) (white bars) or 3-MP (grey bars) followed by addition of 10 nM thrombin (FIIa) for 1 hour. Control wells incubated with FIIa alone for 1 hour (black bars). After a medium change, the supernatants were collected after 48 hours. All conditions performed in triplicate. Graphs represent mean + SD of n=3 experiments.

**Images relate to Figure 5**

**Supplementary figure 6**

A

B

**Supplimentary figure 6: Phenotype of CD68+ cells in response to 3-MP**

Immediately prior to re-challenge with oxazolone, C57BL/6 (WT) mice received either IP saline (n= 8) or 10microM/g IP FLLRN (PAR-1 antagonist (n=8), 2-Furoyl-LIGRLO-amide (PAR- 2 agonist) (n=7), 3-MP (n=6) or 3-MP + FSLLRY-Amide (PAR-2 antagonist (αagonist)) (n=5). Figures show representative three colour IF sections through oxazolone-painted ears. Images show staining with CD68 (red) CD206 (green -A), iNOS (green-B) and DAPI (blue). Dotted lines demarcate the auricular cartilage. AC = auricular cartilage, E= epidermis.

**Images relate to Figure 5**

**Supplimentary figure 7**

A

B

200μm

200μm

C

200μm

**Supplimentary figure 7: Represtntivie IF images in response to PTL0GC1**

C57BL/6 (WT) mice were sensitised on the abdomen with oxazolone on day 0 and re-challenged on day 5 on one of the ears with oxazolone, and on the other ear with vehicle alone. On day 3 and 5 mice received either IV saline (n=4), or 10microM/g IV PTL060 (n=7) or equimolar PTL0GC-1 (n=5) or 3-MP (n=5). 3 hours after injection on day 5 mice were then re-challenged with oxazolone (on the right) or vehicle alone (on the left).

A-C. Representative two colour IF sections through oxazolone-painted ears. Images show staining with ABCA1 (green -A), IL10 (red -B), SOCS3 (green -c) and DAPI (blue). Dotted lines demarcate the internal cartilage. AC = auricular cartilage, E= epidermis.

**Images relate to Figure 6**

**References**

1. Chen D, Xia M, Hayford C, Semik V, Tham E-L, Hurst S, et al. Expression of human tissue factor pathway inhibitor on vascular smooth muscle cells inhibits secretion of macrophage migration inhibitory factor and attenuates atherosclerosis in ApoE-/-mice. Circulation. 2015:CIRCULATIONAHA. 114.013423.

1. Interestingly, thrombin dependent secretion of CCL5 and to a lesser extent CX3CL1 was also partially inhibited by a PAR-2 antagonist, consistent with the notion that a surface PAR cleaved by thrombin was transactivating PAR-2. [↑](#footnote-ref-1)
